# Sirenomelia: A Case Report

**DOI:** 10.31729/jnma.3884

**Published:** 2018-12-31

**Authors:** Pramod Kattel

**Affiliations:** 1 Department of Obstetrics and Gynaecology, B. P. Smriti Hospital, Basundhara, Kathmandu, Nepal

**Keywords:** *case report*, *ectromelia*, *fused legs and feet*, *Mermaid syndrome*, *Sirenomelia*

## Abstract

Sirenomelia is primarily a congenital anomaly where a normally paired lower limb is replaced by a single midline limb and is characterized by single umbilical artery. Such cases though considered rare do occur at our set-up and to make health workers aware regarding the condition, so that they can be managed well when encountered, lays the importance of reporting such case. A referred case of Sirenomelia from Dhading district hospital was presented to Emergency department of Paropakar Maternity and Women's Hospital on 6^th^ March 2016 of 18 year “Young Primigravida at 34 week and 5 days of gestation in second stage of labor” following ultrasonography diagnosis for better management. After confirming the diagnosis, preterm vaginal delivery was performed with a live baby of 1250 gm consisting of multiple congenital anomalies and poor Apgar score. Such cases do occur at our set-up so that if anomaly scanning is done routinely, they could be picked up early and management becomes easier.

## INTRODUCTION

Sirenomelia also known as Sirenomelia sequence or Mermaid syndrome is a rare and fatal anomalous presentation with incidence of 0.98 to 4.2 per 100000 live birth.^1–3^ It is characterized by varying degrees of lower limb fusion associated with other anomalies such as renal agenesis, ano-rectal atresia, agenesis of external genitalia and single umbilical artery.^1,2,4^ They are more common in males with male to female ratio being 2.7:1.^2,4,5^ In term “Sirenomelia” (Latin-Siren + Melia) “Siren” means “a partly female creature of Greek legend” and “Melia” means “limb”.^5,6^ The other term Mermaid sequence is derived from word “Mermaid” (Mere-sea + maid-girl/young woman) which means legendary aquatic creature with the head and upper body of a human female and the tail of a fish.^7,8^ It has resemblance to the mermaid of ancient Greek mythology hence named as such.^9^

## CASE REPORT

Mrs Bhujel, 18 year Young Primigravida presented to Emergency department of Paropakar Maternity and Women's Hospital (PMWH) on 6^th^ March 2016 at 34 week and 5 days of gestation by last menstrual period with obstetric scan done at Dhading district hospital on 24^th^ February 2016 showing viable anomalous fetus with Oligohydramnios. Patient was counseled regarding status of fetus and possible outcomes. Following this, patient was discharged and referred to PMWH where she presented 11 days later only in second stage of labour.

On examination, her general condition was good with no fresh complaints and perceiving good fetal movement. Her general examinations were normal with stable vitals and normal systemic examination. Abdominal examination revealed 34 weeks sized uterus in longitudinal lie with cephalic presentation.

Bedside screening obstetric scan was performed on same day at PMWH which showed viable anomalous baby in cephalic presentation of 35^+^ week of gestation with anterior placentation and scanty liquor. Vaginal delivery was allowed with careful observation with diagnosis of “Primi at 34 weeks and 5 day of gestation with multiple fetal anomalies in second stage of labor”. Spontaneous preterm delivery occurred with findings of polymalformative baby of weight 1250 gm and Apgar score of 2/10, 3/10.

**Figure 1.**
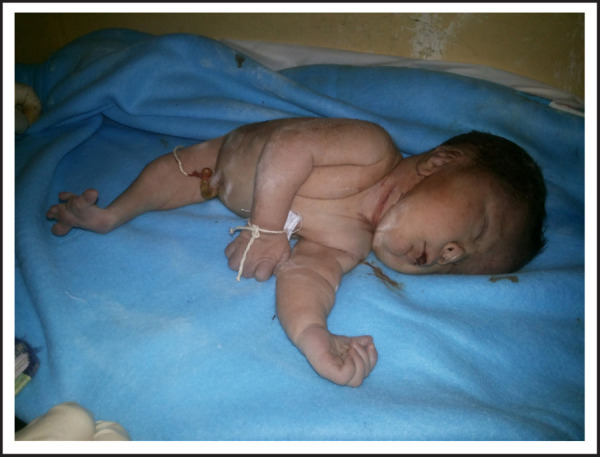
Baby affected with Sirenomelia.

**Figure 2.**
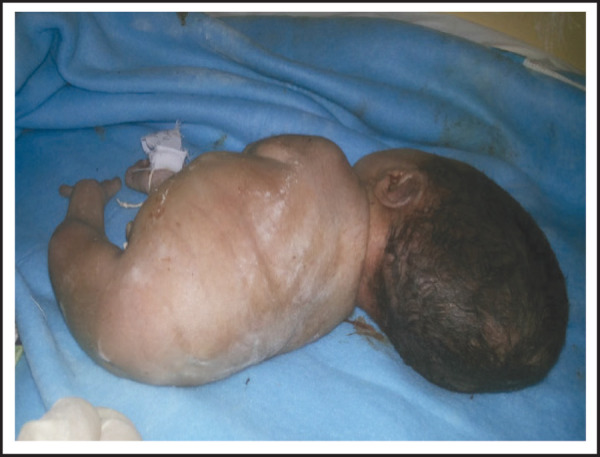
Baby affected with Sirenomelia.

Gross examination of neonate showed Potter facies, dolichocephaly, single umbilical artery, absent external genitalia, imperforate anus, and fused lower limbs and fused feet with distinct plane of fusion-Symelia dipus. The fused feet were rotated with five toes (in total) aligned abnormally resembling fins (Figure 1,2).

Ultrasound abdomen and pelvis showed right renal agenesis, hypoplastic left kidney and non-visualisation of urinary bladder and gonads. Infantogram showed single femur, two tibia and single but partial fibula. Agenesis of sacrum and coccyx were noted.

With oxygen supplementation baby was transferred to neonatal unit and patient party signed for do not resuscitate status with agreement for continued oxygen supplementation and baby expired on same day at seven hour of life.

## DISCUSSION

The risk factors involved are maternal diabetes, tobacco consumption, heavy metal exposure such as Lead, Cadmium, maternal drug abuse like Cocaine, and intake of drugs like Lamotrigine, Retinoic acid, Cyclophosphamide during periconceptional period and the risk decreases with advancing maternal age.^3,7^ Its exact etiology is not known but various theories have been proposed for its genesis.^2,3^ Some of which are vascular steal hypothesis, defective blastogenesis hypothesis and the pressure theory whereas some consider it to be a most severe form of Caudal Regression Syndrome.^2,4^ Embryologically, it occurs due to caudal blastemal defect with persistence of vitelline artery.^7^ Sirenomelia is classified based on lower limb bones i.e. femur, tibia and metatarsal bones.^2^

Sirenomelia is identified using ultra-sonography at early-pregnancy by careful examination of lower limbs.^1^ In the first and early second trimesters, amniotic fluid is sufficient to allow detection of abnormal lower limbs, undetermined external genitalia, anorectal atresia and lumbosacral agenesis. An early antenatal diagnosis is suspected in the presence of Oligohydramnios, bilateral renal agenesis/dysgenesis, malformed lower limbs and a single umbilical artery.^2,4,5^ Abdominal ultrasound shows abnormalities of internal organs. Bilateral renal agenesis cause severe Oligohydramnios hindering a reliable sonographic evaluation of lower extremities in second and third trimesters. Oligohydramnios is sonographic marker of absent or non-functioning kidneys from second half onwards. In such case of diminished amniotic fluid and increased fetal crowding, Magnetic resonance imaging (MRI) will be the investigation of choice. In some cases, bilateral renal agenesis may be the only antenatal sonographic finding and diagnosis is made after termination of pregnancy. Doppler flow imaging reveals umbilical cord with just two vessels. Infantogram performed in post delivery stage shows bony abnormalities. Post-mortem autopsy helps in confirmation of abnormalities or absence of internal organs.

Early diagnosis allows termination of pregnancy at an early stage conferring minor risks and discomfort to the patient.^5,7^ Most cases have premature birth with birth weight less than 2500 gm.^2^ As it is a fatal condition, death usually occurs in one to two day following birth.^1,2,4,7^ Lethality is generally due to obstructive renal failure following renal agenesis/dysgenesis or pulmonary hypoplasia leading to respiratory failure.^4^ So termination of pregnancy is usually opted when diagnosed though there are few cases with survival as well.^7^ Survival depends on nature of visceral abnormalities and development of renal system. About 50% cases born after eight or nine month of pregnancy are alive.

Managing cases of Sirenomelia is difficult and costly. It requires multidisciplinary surgical approaches involving a team of specialists. Among some reported cases of surviving infants with Sirenomelia, “Tiffany Yorks” was the longest (27 years) surviving patient till date and she had undergone successful surgery before age of one year to separate her legs. The other was “Shiloh Pepin” who had lower extremities fused, no bladder, no uterus, no rectum but with one quarter of kidney and one ovary and she survived for 10 years without undergoing surgery to separate lower limbs. The next surviving “Mermaid lady” is “Milagros Cerron” of Peru, who is still alive for more than 14 years and she had undergone successful separation of limbs in two settings.^9,10^

Sirenomelia is more common in males but in this case no sex differentiation could be made. Regular ante-natal check up and anomaly scanning helps in early diagnosis so that timely termination of pregnancy could be done with minimal physical and psychological trauma to pregnant mother. Although it cannot be prevented but proper management can be done once diagnosis is made which is aided by easy availability of MRI at times of diagnostic dilemma usually on later weeks of gestation and during severe Oligohydramnios which is the usual fate of such fetuses.

Such rare cases may be faced by anyone at any centre at any time. Proper counseling to the patient and patient party should be provided regarding management plan, their pros and cons and their chances of recurrence in future pregnancies. Our plan of management should depend on gestational age, condition of fetus, availability and feasibility of treatment options along with wish of parents.
